# Fluid shear stress regulates the survival of circulating tumor cells via nuclear expansion

**DOI:** 10.1242/jcs.259586

**Published:** 2022-05-26

**Authors:** Zichen Xu, Keming Li, Ying Xin, Kai Tang, Mo Yang, Guixue Wang, Youhua Tan

**Affiliations:** ^1^ The Hong Kong Polytechnic University Shenzhen Research Institute, Shenzhen 518000, China; ^2^ Research Institute for Smart Ageing, The Hong Kong Polytechnic University, Hong Kong 999077, China; ^3^ Department of Biomedical Engineering, The Hong Kong Polytechnic University, Hong Kong 999077, China; ^4^ Key Laboratory for Biorheological Science and Technology of Ministry of Education, State and Local Joint Engineering Laboratory for Vascular Implants, Bioengineering College of Chongqing University, Chongqing 400030, China

**Keywords:** Fluid shear stress, Mechanobiology, Circulating tumor cell, Histone acetylation, Nuclear size

## Abstract

Distant metastasis mainly occurs through hematogenous dissemination, where suspended circulating tumor cells (CTCs) experience a considerable level of fluid shear stress. We recently reported that shear flow induced substantial apoptosis of CTCs, although a small subpopulation could still persist. However, how suspended tumor cells survive in shear flow remains poorly understood. This study finds that fluid shear stress eliminates the majority of suspended CTCs and increases nuclear size, whereas it has no effect on the viability of adherent tumor cells and decreases their nuclear size. Shear flow promotes histone acetylation in suspended tumor cells, the inhibition of which using one drug suppresses shear-induced nuclear expansion, suggesting that shear stress might increase nuclear size through histone acetylation. Suppressing histone acetylation-mediated nuclear expansion enhances shear-induced apoptosis of CTCs. These findings suggest that suspended tumor cells respond to shear stress through histone acetylation-mediated nuclear expansion, which protects CTCs from shear-induced destruction. Our study elucidates a unique mechanism underlying the mechanotransduction of suspended CTCs to shear flow, which might hold therapeutic promise for CTC eradication.

## Teaser

**Summary:** This study reports that suspended tumor cells respond to fluid shear stress through histone acetylation-mediated nuclear expansion, which protects CTCs from shear-induced apoptosis.

## INTRODUCTION

Metastasis is a complex and sequential process, mainly involving cell detachment from the primary lesion, local invasion, intravasation into and survival in the vasculature, extravasation into other organs, and formation of secondary tumors (Valastyan and Weinberg, 2011). Tumor cells metastasize to distant organs mainly through hematogenous dissemination (Alix-Panabières and Pantel, 2014), in which cell survival is one determinant factor of metastasis inefficiency. A high frequency of circulating tumor cells (CTCs) in the vasculature is correlated with poor prognosis and patient survival (Alix-Panabières and Pantel, 2014). After entering the circulation, tumor cells stay in suspension and exhibit substantial apoptosis, although a subpopulation of CTCs persist and have resistance to the destruction mediated by blood circulation, and these cells might eventually grow into metastatic tumors (Alix-Panabières and Pantel, 2014; Valastyan and Weinberg, 2011). Metastases account for over 90% of cancer deaths and become the grand challenge in cancer therapeutics (Valastyan and Weinberg, 2011). Therefore, unveiling the critical factors underlying the survival of CTCs during hematogenous dissemination is important to facilitate the development of new therapeutic strategies for eradicating CTCs and preventing metastasis.

Except for the biochemical mechanisms underlying their intravascular survival (Alix-Panabières and Pantel, 2014), CTCs experience considerable levels of fluid shear stress in the vasculature, which could be a key rate-limiting factor for tumor cell survival, since mechanical cues regulate cellular functions and play important roles in tumor metastasis (Wirtz et al., 2011). The influence of fluid shear stress on the survival of tumor cells that adhere to solid substrates (adherent cells) has been extensively investigated (Follain et al., 2020; Huang et al., 2018) – high shear stress promotes the production of reactive oxygen species and induces damage and apoptosis in lung cancer cells (Lo et al., 2013); fluid shearing sensitizes cancer cells to radiation-induced apoptosis via integrin and focal adhesion kinase (Luo et al., 2014); low shear stress reduces the viability of ovarian cancer cells (Hyler et al., 2018); laminar shear flow induces the autophagy and apoptosis of various types of cancer cells (Lien et al., 2013); and adherent tumor cells sense fluid shear stress through the transient receptor potential channel melastatin 7, which enhances Ca^2+^ influx and reverses cell migration direction (Yankaskas et al., 2021). In comparison, the effect of shear stress on tumor cells in suspension remains less understood – the viability and proliferation of suspended colon CTCs depend on shear stress and circulation time (Fan et al., 2016); shear flow sensitizes suspended colon and prostate tumor cells to apoptosis (Mitchell and King, 2013); high shear stress induces significant apoptosis of CTCs (Regmi et al., 2017) and facilitates migration and extravasation (Ma et al., 2017); circulatory fluid stress leads to the reprogramming of gene expression pattern and enhances metastatic potential of lung cancer cells (Alvarado-Estrada et al., 2021). Nevertheless, malignant tumor cells exhibit resistance to the destruction of shear stress (Barnes et al., 2012; Hope et al., 2021), which depends on the expressions of oncogenes and lamin A/C (Mitchell et al., 2015). Our previous work reports that fluid shear stress influences the viability and adhesion of suspended CTCs and enhances their drug resistance ability (Xin et al., 2019). Shear flow activates the c-Jun N-terminal kinase signaling and epithelial–mesenchymal transition and reduces F-actin and cellular stiffness, both of which affect the survival of suspended tumor cells in shear flow (Xin et al., 2019; Xin et al., 2020). Furthermore, fluid shear stress upregulates the expressions of the genes related to brain metastasis and stemness (Jin et al., 2018). Therefore, both adherent and suspended tumor cells sense and respond to fluid shear stress. However, the mechanotransduction of suspended tumor cells and the mechanisms underlying the resistance of CTCs to blood shear flow are still not clear.

Nuclear size is closely linked to chromatin organization and compaction, which are critical factors in the regulation of the accessibility and transcription of many genes (Jevtić et al., 2014). Therefore, the change of nuclear size is important in various cellular functions, including proliferation, differentiation and development (Webster et al., 2009). Aberrant alteration of nuclear size is associated with many diseases, including cancer (Zink et al., 2004). Various factors regulate nuclear size, including DNA content and nuclear structural elements (Webster et al., 2009). Recent findings show that mechanical cues impact nuclear size – substrate stiffness, spreading area, and osmotic pressure strongly influence cell and nuclear volume, which affects stem cell properties (Guo et al., 2017); mechanical compression and stretching reduces and increases nuclear size of embryonic stem cells, respectively (Pagliara et al., 2014); fluid shear stress reduces the cell and nuclear volume of osteoblasts and upregulates the adhesion and structural proteins (Jin et al., 2020); shear flow induces the nuclear shrinkage of epithelial cells through Piezo1-mediated Ca^2+^ influx (Jetta et al., 2019). All these findings demonstrate that nuclear size is sensitive to mechanical stimulations in adherent cells. However, it remains unclear whether tumor cells in suspension alter their nuclear volume in response to fluid shear stress and how the alteration in nuclear size influences the mechanotransduction and survival of suspended CTCs under shear flow.

In this study, suspended breast tumor cells were circulated under various magnitudes of fluid shear stress. The survival and cell and nuclear size of the treated cells were analyzed. To explore the mechanism underlying nuclear expansion, histone acetylation of shear-treated tumor cells and the expressions of histone acetyltransferases (HATs) were measured. The influence of HAT activity on shear-induced increase in nuclear size was demonstrated. Finally, the role of shear-induced nuclear expansion in the survival of suspended CTCs under shear flow was examined.

## RESULTS

### Fluid shear stress reduces the survival and increases the nuclear size of suspended but not adherent tumor cells

**Fig. 1.**
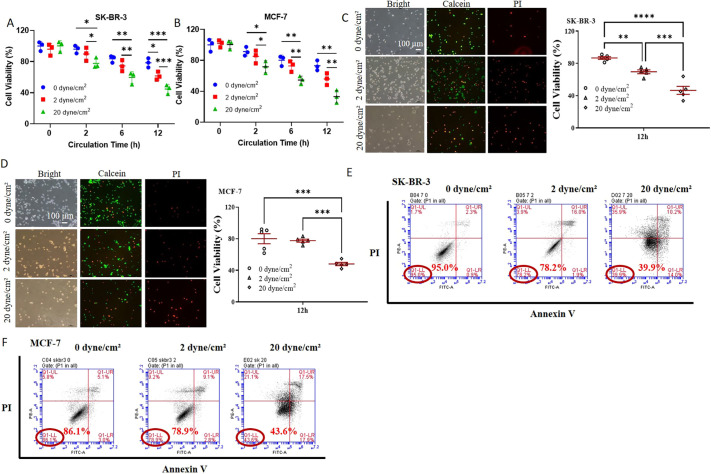
**Fluid shear stress eliminates the majority of suspended tumor cells in a magnitude-dependent manner.** Breast cancer cells SK-BR-3 (A,C,E) and MCF-7 cells (B,D,F) were circulated under 0, 2, and 20 dyne/cm^2^ shear stress for 0, 2, 6 and 12 h (A,B) and 12 h (C–F), respectively, after which cell viability was measured by means of an MTS assay (A,B), Calcein AM/PI staining (C,D), and Annexin V apoptosis assay (E,F). Representative images are shown on the left panels of C,D and the results are quantified in the right panels (*n*=3). Results in E and F are representative of two experiments. *P<0.05; **P<0.01; ***P<0.001 (two-way ANOVA with post-hoc Bonferroni test). All quantitative data is mean±s.e.m.

To explore the influence of fluid shear stress on CTCs in suspension, an *in vitro* microfluidic system was developed previously to mimic the shear flow in blood circulation (Fig. S1A) (Jin et al., 2018; Xin et al., 2019), where fluid shear stress and circulation duration were within 20 dyne/cm^2^ and 12 h, respectively (Alix-Panabières and Pantel, 2014; Fan et al., 2016). The fluid shear stress reported in this study represents the stress on the tube wall or wall shear stress. To test the effect of shear flow on tumor cell survival, single breast cancer cells were circulated in suspension under various magnitudes of wall shear stress and circulation time in the microfluidic system. Cell viability was measured by MTS assay, Calcein-acetoxymethyl (AM)/Propidium iodide (PI) staining, and Annexin V assay. The results show that the survival of both suspended SK-BR-3 and MCF-7 cells was gradually reduced from 100% to ∼40% or 30% along with the increase of wall shear stress from 0 to 20 dyne/cm^2^ and the time from 0 to 12 h (Fig. 1). These findings suggest that suspended tumor cells have the ability to sense and respond to fluid shear stress, which is consistent with our previous work (Xin et al., 2019, 2020). In comparison, when tumor cells were attached to solid substrates (adherent cells), fluid shear stress within 0.6 dyne/cm^2^ had no obvious effect on cell survival after 12 h treatment (Fig. S1C–E). Note that wall shear stress higher than 0.8 dyne/cm^2^ detached SK-BR-3 or MCF-7 cells notably from the underlying substrate.

**Fig. 2.**
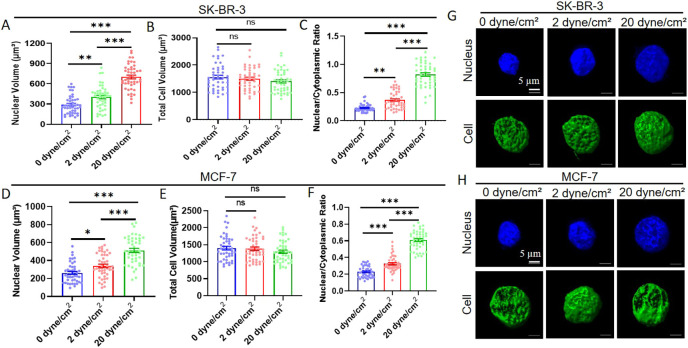
**Fluid shear stress induces considerable nuclear expansion in suspended tumor cells.** SK-BR-3 (A–C,G) and MCF-7 (D–F,H) cells were circulated under 0, 2 and 20 dyne/cm^2^ shear stress for 12 h, respectively. The cell cytoskeleton and nuclei of these treated cells were stained for the measurement of nuclear (A,D) and cell volume (B,E) by confocal imaging. The nuclear to cytoplasmic ratio was then calculated (C,F). Representative 3D reconstructed images of cells and nuclei are shown in G,H. *n*>45 cells. *P<0.05; **P<0.01; ***P<0.001; ns, not significant (one-way ANOVA with post-hoc Bonferroni test). All quantitative data is mean±s.e.m.

We further stained the cytoskeleton (F-actin) and nucleus of shear-treated suspended tumor cells and measured the volumes of the whole cell and nucleus by confocal imaging. The results show that fluid shear flow remarkably increased the nuclear volume of suspended tumor cells by 2–2.5-fold in a magnitude-dependent manner (Fig. 2A,D,G,H). Interestingly, cell volume was not significantly affected by shear stress (Fig. 2B,E,G,H). As a result, shear flow enhanced the ratio of nuclear and cytoplasmic volume by 2.5–4-fold (Fig. 2C,F). In contrast, fluid shear stress moderately decreased the nuclear volume of adherent tumor cells (Fig. S1F,I,J,M) but did not affect the whole cell size (Fig. S1G,I,K,M), and therefore significantly reduced the ratio of nuclear and cytoplasmic volume by ∼20% (Fig. S1H,L). In addition, fluid shear flow affected the nuclear morphology, increased the nuclear circularity, and decreased the aspect ratio of both suspended and adherent tumor cells (Fig. S2A,B,D,E,G–K). All these results demonstrate that fluid shear stress reduces the survival and increases nuclear size of suspended tumor cells, whereas shear flow does not influence the viability but slightly decreases the nuclear size of adherent tumor cells, which together suggest that there is a differential mechanoresponse of adherent and suspended tumor cells to fluid shear stress.

### Fluid shear stress enhances nuclear size of suspended circulating tumor cells via histone acetylation

**Fig. 3.**
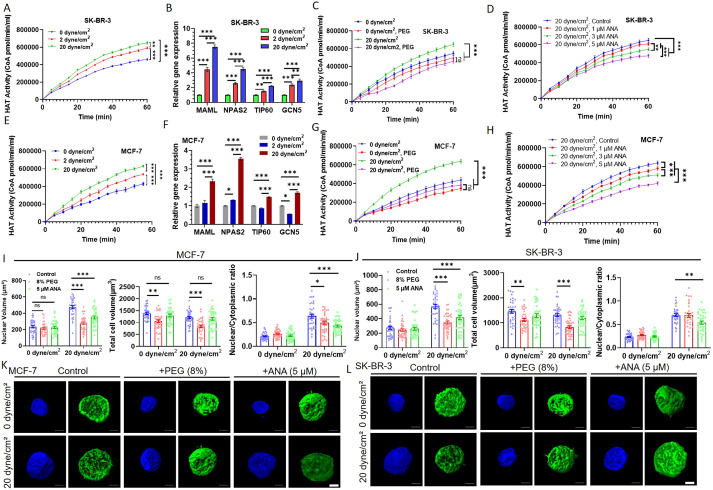
**Fluid shear stress increases nuclear size of suspended tumor cells via upregulated HAT activity.** (A,E) Fluid shear stress enhances the HAT activity of suspended tumor cells. SK-BR-3 (A) and MCF-7 (E) cells in suspension were circulated under 0, 2 and 20 dyne/cm^2^ shear stress for 12 h, respectively. The HAT activity of these treated cells was measured by means of the HAT activity assay kit at the indicated time points. *n*=4. (B,F) Fluid shear stress upregulates the expression of HATs and HAT-associated genes in suspended tumor cells. The mRNA levels of genes of the cells treated as in A,E, including *MAML1* (MAML), *NPAS2*, *TIP60* and *GCN5*, were measured by quantitative RT-PCR. *n*=3. (C,D,G,H) Osmotic stress and HAT inhibitors block the shear-induced increase in HAT activity of suspended tumor cells. SK-BR-3 (C,D) and MCF-7 (G,H) cells in suspension were circulated under 0 and 20 dyne/cm^2^ shear stress in the presence and absence of 8% PEG and various doses of HAT inhibitor ANA (1, 3 and 5 µM), respectively. Their HAT activity was measured. *n*=4. (I,J) Inhibiting HAT activity attenuates shear-stress-induced nuclear expansion in suspended tumor cells. Suspended MCF-7 (I) and SK-BR-3 (J) cells were circulated under 0 and 20 dyne/cm^2^ shear stress in the presence and absence of 8% PEG and 5 µM ANA, respectively. The nuclear and cell size of these treated cells were measured. *n*>35 cells. (K,L) Representative images of the nucleus and cell of the cells in I,J. Scale bars: 5 μm. *P<0.05; **P<0.01; ***P<0.001; ns, not significant (two-way ANOVA with post-hoc Bonferroni test). All quantitative data is mean±s.e.m.

Nuclear size is associated with chromatin organization (Mukherjee et al., 2016). Low chromatin compaction usually loosens the interaction between histones and DNA and leads to high gene accessibility and thus large nuclear size, whereas high chromatin condensation suppresses gene transcription and shrinks the nucleus. One important mechanism that affects chromatin compaction and nuclear size is histone acetylation (Görisch et al., 2005), which depends on the activity of HATs. We hypothesize that fluid shear stress increases nuclear size of suspended CTCs through the upregulation of histone acetylation. To test this possibility, suspended breast cancer cells were subjected to various shear stresses and their HAT activity was measured accordingly and utilized to represent the level of histone acetylation. The results show that HAT activity was enhanced from 0, 2 to 20 dyne/cm^2^ shear stress by 41% and 46% for SKBR-3 and MCF-7 cells, respectively (Fig. 3A,E), which might be explained by the upregulation of HATs and the associated genes [e.g. MAMLs (e.g. *MAML1*), NPASs (e.g. *NPAS2*), *TIP60* (also known as *KAT5*) and *GCN5* (also known as *KAT2A*); Fig. 3B,F; Fig. S3]. Since osmotic stress significantly affects histone acetylation (Irianto et al., 2013; Lima et al., 2018), we further examined the influence of osmotic stress on shear-induced HAT activity. Consistent with our above hypothesis, 20 dyne/cm^2^ shear stress induced higher HAT activity than 0 dyne/cm^2^ shear stress, whereas elevating osmotic stress through treatment with 8% polyethylene glycol (PEG) diminished this shear-induced increase in HAT activity (Fig. 3C,G). To test the role of upregulated HATs in the enhanced HAT activity, anacardic acid (ANA), a potent HAT inhibitor, was used to treat suspended tumor cells under 0 and 20 dyne/cm^2^ shear flow. Inhibiting HATs reduced the shear-induced HAT activity of suspended cells under 20 dyne/cm^2^ shear flow in a dose-dependent manner (Fig. 3D,H). Note that similar treatment had a very small effect on the HAT activity of suspended cells under 0 dyne/cm^2^ shear stress (Fig. S4A,B). ANA and PEG had no significant cytotoxicity on breast cancer cells (Fig. S4C,D). All these findings demonstrate that fluid shear stress facilitates HAT activity and histone acetylation of suspended tumor cells via the upregulation of HATs.

To further explore the role of the elevated HAT activity in shear-induced increase in nuclear size, suspended tumor cells were circulated under 0 and 20 dyne/cm^2^ shear stress for 12 h, while their HAT activity was inhibited by 8% PEG or 5 µM ANA. The measurement of the cell and nuclear size of these treated cells shows that inhibiting HAT activity by both PEG and ANA reduced nuclear volume significantly and cell volume moderately, and thus notably decreased the ratio of nuclear to cytoplasmic volume of suspended tumor cells under 20 dyne/cm^2^ shear stress (Fig. 3I–L). This finding suggests that the treatment preferentially reduces the nuclear volume. Nevertheless, it is better to specifically inhibit the nuclear but not cell size under fluid shear stress to explore the influence of nuclear size. Note that similar treatment did not significantly affect the nuclear size and the ratio of nuclear and cytoplasmic volume of suspended cells but slightly reduced the cell volume under 0 dyne/cm^2^ shear stress (Fig. 3I–L). PEG and ANA treatment diminished the shear-induced difference in nuclear circularity between 0 and 20 dyne/cm^2^ shear stress (Fig. S2C,F). These results suggest that osmotic stress and inhibition of HAT activity might specifically block shear-induced increase in nuclear size. In summary, all these findings demonstrate that fluid shear stress elevates the nuclear size of suspended CTCs via an increase in HAT activity.

### Shear-induced nuclear expansion protects suspended tumor cells from the destruction of fluid shear stress

**Fig. 4.**
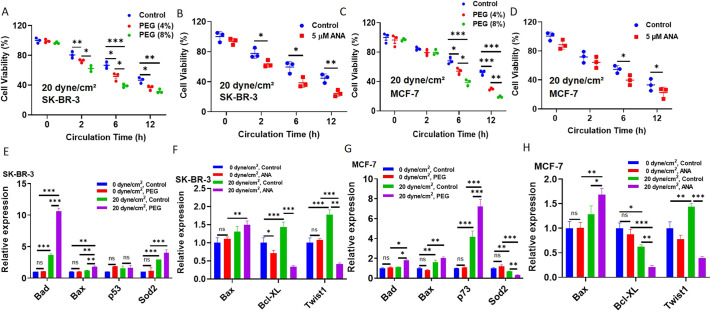
**The shear-induced nuclear expansion protects suspended tumor cells from the destruction upon fluid shear stress.** (A–D) Inhibiting histone acetylation-mediated nuclear expansion decreases the viability of suspended tumor cells under fluid shear stress. Suspended SK-BR-3 (A,B) and MCF-7 (C,D) cells were circulated under 0 and 20 dyne/cm^2^ shear stress in the presence and absence of 4% and 8% PEG or 5 µM ANA for 0, 2, 6 and 12 h, respectively, when cell viability was measured by MTS assay. *n*=3. (E–H) Inhibiting histone acetylation-mediated nuclear expansion upregulates the expressions of pro-apoptosis genes and downregulates the expressions of anti-apoptosis genes. The expressions of pro-apoptosis and anti-apoptosis genes in the cells treated as in A–D were measured by quantitative RT-PCR. *n*=3. *P<0.05; **P<0.01; ***P<0.001; ns, not significant (two-way ANOVA with post-hoc Bonferroni test). All quantitative data is mean±s.e.m.

We further explored the influence of shear-induced nuclear expansion on the survival of suspended tumor cells under fluid shear stress. To address this issue, suspended breast cancer cells were treated under 20 dyne/cm^2^ shear stress in the presence of osmotic stress (4% and 8% PEG) and the HAT inhibitor ANA (5 µM), respectively, which can suppress the shear-induced nuclear expansion. Inhibiting the HAT-mediated increase in nuclear size by means of PEG significantly reduced the survival of suspended CTCs under shear flow by ∼32–60% in a dose-dependent manner for both SKBR-3 and MCF-7 cells (Fig. 4A,C). Similarly, inhibiting HAT activity with ANA decreased cell viability notably, by 33–44% (Fig. 4B,D). In addition, the combination of PEG and ANA further reduced cell viability to an even lower level (Fig. S4E). PEG and ANA had no significant influence on the survival of suspended MCF-7 and SKBR-3 cells under 0 dyne/cm^2^ shear stress (Fig. S4C,D), suggesting that the decrease in tumor cell viability is not due to the pro-apoptosis side effect of the drug treatment. The addition of PEG might increase the medium viscosity and thus fluid shear stress. To maintain the same wall shear stress when PEG is added, the flow rate needs to be adjusted correspondingly according to the Poiseuille's law. Our results show that the addition of PEG reduced cell survival compared to control and there was no significant difference in cell viability between the control and adjusted flow rate (Fig. S4F,G), suggesting that PEG decreases cell survival under shear flow not through the effect on medium viscosity and shear stress. Together with the fact that inhibiting histone acetylation blocks shear-induced increase in nuclear size (Fig. 3I–L), these findings indicate that shear-induced nuclear expansion via histone acetylation might protect suspended CTCs from shear-mediated destruction.

To further explore the underlying mechanism, the influence of histone acetylation on the expression of the genes related to cell apoptosis/survival was examined. When suspended tumor cells were circulated under 0 dyne/cm^2^ shear stress, treatment with PEG and ANA had no significant effect on the expression of the genes related to pro-apoptosis and anti-apoptosis (Fig. 4E–H), which might explain their minimal influence on cell viability. In contrast, inhibiting histone acetylation by PEG or ANA notably upregulated the expression of pro-apoptosis genes (e.g. Bad, Bax, p53 and p73) while downregulating the expression of anti-apoptosis genes (e.g. Sod2, Bcl-XL and Twist1) under the treatment of 20 dyne/cm^2^ shear stress (Fig. 4E–H). The reduced expression of anti-apoptosis genes and elevated expression of pro-apoptosis genes might lead to a significant reduction in tumor cell survival upon shear stress, which could partially explain the effect of inhibiting histone acetylation on tumor cell survival.

## DISCUSSION

The metastatic efficiency of disseminated tumor cells is rather low (e.g. less than 0.01%) mainly due to the existence of various rate-limiting factors in the microenvironment that cells encounter during the metastasis process (Alix-Panabières and Pantel, 2014; Kang and Pantel, 2013; Valastyan and Weinberg, 2011). In particular, when tumor cells intravasate and enter into the vasculature, CTCs lose the anchorage to solid substrates and the protection from the primary tumor microenvironment. Instead, they become suspended and vulnerable to many biochemical (e.g. cytokines, immune cells) and biophysical cues (e.g. fluid shear stress) in the circulation system. Our current and previous work, together with many others, has shown that CTCs in suspension are able to sense and respond to fluid shear stress (Fan et al., 2016; Jin et al., 2018; Mitchell and King, 2013; Regmi et al., 2017; Xin et al., 2019; Xin et al., 2020). As a result, blood shear flow can induce substantial CTC apoptosis. Nevertheless, a minor subpopulation of CTCs persist and survive in the vasculature and might contain the metastasis-initiating cells with the ability to eventually generate the metastatic tumors. The mechanotransduction mechanisms of adherent tumor cells in response to various mechanical cues have been extensively studied (Broders-Bondon et al., 2018). In contrast, how suspended CTCs respond to and survive under fluid shear stress remains poorly understood.

This study reports that fluid shear stress reduces the viability of CTCs in suspension and considerably increases the nuclear size of surviving tumor cells. Interestingly, shear flow slightly reduces the nuclear size of adherent tumor cells. These findings suggest that nuclear expansion is one unique mechanotransductive response of suspended cells to fluid shear stress and that the status of suspension or adherence affects the mechanoresponse of tumor cells to mechanical cues. The difference in the mechanotransduction mechanisms between suspended and adherent tumor cells remains unknown and is worthy of further investigation in the future. Furthermore, fluid shear flow enhances the HAT activity and histone acetylation, which mediate the nuclear expansion of suspended tumor cells. Previous studies have shown that circulatory shear flow increases the nuclear area of suspended SK-BR-3 cells with and without confinement (Cognart et al., 2020). When cells are attached to solid substrates, fluid shear stress activates Piezo1-mediated Ca^2+^ signaling and reduces nuclear size (Jetta et al., 2019). Pulsating fluid flow reduces the cell and nuclear size of osteoblasts (Jin et al., 2020). Nevertheless, shear flow induces H2B acetylation, chromatin decondensation, and nuclear expansion of human embryonic stem cells (Wang et al., 2019).

Nuclear size is related to chromatin organization, which influences gene accessibility and transcription, and thus plays important roles in many cellular functions (Chow et al., 2012; Edens et al., 2013). Nuclear morphology and size are correlated with tumor progression (Chow et al., 2012; Edens et al., 2013). Malignant cells in pancreatic cancer exhibit larger nuclei compared to benign cells (Taira et al., 2012). However, the influence of the nuclear size of CTCs on their survival under fluid shear flow remains unclear. This study reports that shear-induced histone acetylation and nuclear expansion confer suspended tumor cells a survival advantage in blood shear flow and protect CTCs from the destruction of fluid shear stress. It is possible that high histone acetylation mediates lower chromatin compaction and the larger nuclear size, which might loosen the chromatin structure and enhance the accessibility and transcription of the genes related to survival. This new finding unveils a novel mechanotransductive mechanism underlying the resistance of a small subpopulation of CTCs to fluid shear flow. Targeting shear-induced nuclear expansion might serve as a potential therapeutic strategy for CTC eradication and metastasis prevention. Several studies have shown that nuclear size of CTCs is correlated with the metastatic risk and tumor progression (Chen et al., 2017). A larger nuclear size of CTCs is associated with worse clinical outcome in metastatic castration-resistant prostate cancer (Gill et al., 2017), whereas the existence of CTCs with very small nuclei is linked to high risk of visceral metastases and mortality in advanced prostate cancer (Chen et al., 2015; Wang et al., 2021). Therefore, these contradictory findings suggest an unclear relationship between the nuclear size of CTCs and tumor stage/metastatic risk. More importantly, the role of nuclear size in tumor metastasis is largely unknown.

We have demonstrated that suspended CTCs can sense and respond to fluid shear stress in the vasculature through histone acetylation-mediated nuclear expansion, which protects them from shear-induced elimination. However, the mechanotransduction mechanism is not completely understood, especially the mechanosensor by which suspended cells sense fluid shear stress. It is well known that adherent tumor cells can sense mechanical cues through Piezo1 (Dombroski et al., 2021). For example, glioblastoma cells sense matrix stiffness via Piezo1-mediated mechanotransduction (Chen et al., 2018). Fluid shear stress induces nuclear shrinkage in adherent epithelial cells through Piezo1-mediated Ca^2+^ influx (Jetta et al., 2019). Recent evidence shows that fluid shear stress enhances the sensitivity of suspended tumor cells to TRAIL-mediated apoptosis via Piezo1-mediated Ca^2+^ influx and activation of calpains (Hope et al., 2019). Therefore, it is possible that suspended tumor cells might sense fluid shear stress via Piezo1 channel, which might further affect histone acetylation and nuclear size. In the future, it is important to elucidate the role of Piezo1 in the mechanosensing of suspended cells.

Of note, CTCs in cancer patients are very rare (1–10 CTCs per ml blood) (Alix-Panabières and Pantel, 2014; Cristofanilli et al., 2004). As such, cancer cell lines in suspension have been adopted in this study as an alternative model to represent certain aspects, but these do not necessarily represent the bona fide biology of CTCs from cancer patients. Furthermore, this study investigates the influence of fluid shear stress on CTCs, which greatly simplifies the microenvironmental factors in the vasculature (Alix-Panabières and Pantel, 2014; Cristofanilli et al., 2004). In order to extend our current findings to the clinical setting, it is necessary to rigorously test the ideas using patient CTCs expanded using sophisticated techniques in the vascular microenvironment.

### Conclusions

This study reports that fluid shear stress in the vasculature eliminates the majority of suspended CTCs, although a subpopulation of these cells persist and survive under shear flow. Importantly, blood shear flow considerably increases the nuclear size of the surviving cells in suspension while moderately decreases the nuclear size of adherent tumor cells, suggesting that there is a distinct response of suspended and adherent tumor cells to shear flow. The nuclear expansion in suspended CTCs is mediated by a shear-induced increase in HAT activity and histone acetylation. Remarkably, shear-induced histone acetylation and nuclear expansion enable CTCs to evade the destruction of fluid shear stress, possibly by upregulating the transcription of the genes related to survival. In summary, this study unveils a new mechanotransduction mechanism for suspended tumor cells to survive under blood shear flow, targeting of which might provide a novel therapeutic strategy for CTC eradication and metastasis prevention.

## MATERIALS AND METHODS

### Cell culture

Human breast cancer cell lines SK-BR-3 and MCF-7 were purchased from the ATCC (Manassas, VA). SK-BR-3 cells and MCF-7 cells were maintained in Dulbecco's modified Eagle's medium (DMEM; HyClone Laboratories, Logan, UT) and RPMI 1640 medium (HyClone Laboratories) both supplemented with 10% fetal bovine serum (FBS) and 1% penicillin (HyClone Laboratories). Cells were incubated in the cell culture incubator with 5% CO_2_ at 37°C.

### Shear stress treatment

A microfluidic system was constructed using a peristaltic pump (Harvard Peristaltic Pump P-230, Holliston, MA; Ismatec Gear Pump BVP-Z) and micro silicone tubing. This system can generate a pulsatile flow that can simulate hemodynamic shear stress. To treat cells attached to solid substrates, breast cancer cells were cultured on collagen-coated flow chamber chips (μ-Slide I0.6, ibidi, Fitchburg, WI), and subjected to various levels of fluid shear stress. According to the manufacturer's instruction, wall shear stress was calculated by τ=60.1×μQ, where Q is the flow rate and μ is the dynamic viscosity of the fluid (0.01 dyne·s/cm^2^ for cell culture medium). When tumor cells were in suspension, wall shear stress τ (dyne/cm^2^) in the tubing was calculated by τ=4μQ/(πR^3^) according to Poiseuille's law, where R is the tubing radius (R=0.255 mm). The whole system was sterilized by 75% ethanol before experiments, then rinsed with 4 ml phosphate-buffered saline (PBS; HyClone Laboratories) and 4 ml 1% bovine serum albumin (VWR Life Science, Radnor, PA). Cell suspension solution (2×10^5^ cells/ml) was added into the circulatory system and subjected to various magnitudes of shear stress for different durations (0–20 dyne/cm^2^; 0–12 h) in 5% CO_2_ at 37°C.

### Drug treatment

Cells were treated with 8% (w/w) 300-Da polyethylene glycol (PEG 300, P103728, Aladdin, CHN), and 1, 3 and 5 μM anacardic acid (ANA, ab120892, Abcam, UK). The drug was present throughout the cycle of shear stress treatment.

### MTS assay

Cell viability was measured with an MTS assay (Promega, Madison, WI) following the manufacturer's protocol. In brief, 100 μl of cell suspension was collected from the circulatory system and then added into one well of a 96-well plate. After 12 h of incubation, 20 μl of sterilized CellTiter 96 Aqueous One Solution (5 mg/ml; Promega) was added to each well, and the plate was incubated at 37°C for 4 h in dark. The absorbance of the cell solution was measured at 492 nm using a microplate reader (Ledetect 96, Labexim Products, Lengau, Austria).

### Confocal imaging for the measurement of cell and nuclear volume

After shear stress treatment, the suspended cells were collected in a centrifuge tube and washed with PBS. Then these cells or tumor cells cultured on the flow chamber chip were fixed with 4% paraformaldehyde in PBS for 15 min at room temperature, permeabilized with 0.1% Triton X-100 for 5 min, and blocked with 1% BSA for 1 h. Cells were stained for F-actin (ab112125, Abcam) and with DAPI (11569306, Invitrogen, USA) for 1 h at room temperature. After being washed three times in PBS with 0.1% Triton X-100 for 5 min, samples were visualized under a confocal laser scanning microscope with a 63×/1.2 NA oil objective (TCS SPE, Leica, GER). *Z*-stack images with 1024×1024 pixels were collected at a 0.3 μm slice interval, stepping through the entire cell.

The confocal *z*-stack images were analyzed using ImageJ (NIH, USA) and Imaris (Oxford Instruments, UK) software. For the volume measurement, image stacks were input into Imaris in their original format and automatically reconstructed into a multi-channel 3D model. To designate individual cancer cells of interest, the ‘Surface’ creation tool was used to generate a region of interest (ROI). In order to create a clean boundary around the desired cell, surface details were smoothed using surface particles, depending on the shape of individual cells and the distribution of early endosomes in the cytoplasm. In the Slice Mode, cell width was measured at the widest point of the cell. This measurement was rounded to the nearest integer and entered as the diameter of the largest sphere in the Surface creation. To quantify the nuclear volume and cell volume, the ‘Spot’ creation tool was used. In the Spot creation wizard, different spot sizes were selected to allow measurements of endosomes in different diameter ranges. The *xy* value for spot detection was estimated to be 0.178 μm, and the *z*-value was determined by the interval between layers. During spot detection, the area growth diameter was measured by measuring the area volume. For the 3D reconstruction, *z*-stacks were opened in ImageJ in their original format. The split blue and green channels were used to convert the image from a RGB stack to a composite image. Individual slices comprising the entire z-stack were compiled into a 3D image using the plugin 3Dscript. To limit the vesicle measurement to individual cells of interest, we plotted a ROI along the perimeter of the cell using a freehand selection tool.

### PI staining

For adherent tumor cells, PI staining was used to measure cell viability following the manufacturer's instructions. In brief, tumor cells after shear stress treatment were rinsed with PBS three times. The stock solution of PI (1 mg/ml or 1.5 mM, Thermo Fisher Scientific, Waltham, MA, USA) was diluted (1:3000) into 500 nM working solution in PBS. Tumor cells were then incubated with the PI working solution for 5 min at 37°C. The cells were imaged under a fluorescence microscope (Nikon, Tokyo, Japan). At least 150 cells were imaged for each condition.

### Annexin V/PI assay

To measure the apoptosis of suspended tumor cells, the Annexin V-fluorescein isothiocyanate (FITC) Apoptosis Staining/Detection kit (ab14085, Abcam, Cambridge, UK) and BD Accuri C6 Flow Cytometer (BD Biosciences, San Jose, CA, USA) were used. We collected and re-suspended 100,000 cells in 500 µl of binding buffer (Abcam, Cambridge, UK). 5 µl of Annexin V–FITC and PI were added to the suspended cells, and cells were incubated for 5 min without light at 4°C. Then, a flow cytometer was used to fractionate at least 10,000 cells by detecting fluorescence from FITC and phycoerythrin signals, and the results were analyzed using BD Accouri C6 software.

### Calcein-AM and PI assay

To quantify the percentages of live and dead cells, suspended tumor cells were stained with Calcein-AM and PI. Suspended tumor cells were collected by centrifugation for 5 min (200 ***g***), washed with PBS and incubated in a PBS solution containing 2 µl Calcein-AM and 4 µl PI for 30 min at room temperature. The cells were resuspended in PBS and imaged with a Leica confocal microscope. To determine the proportion of living cells, we calculated the number of Calcein+ and PI− suspended cells using ImageJ.

### Histone acetyltransferase activity assay

Following shear stress treatment, the cells were collected and then nuclear extract was obtained by using Nuclear Extraction Reagents (#78833, Thermo Fisher Scientific, USA). HAT activity was measured with the HAT activity assay kit (ab239713, Abcam) following the manufacturer's protocol. In brief, the standard sample, background controls and positive controls were prepared according to the instructions, and added with reaction mix reagents into a 96-well plate. Fluorescence signals (excitation, 535 nm; emission, 587 nm) were read by using a multimode microplate reader (Varioskan LUX, Thermo Fisher Scientific) in the kinetic mode at 30°C for 60 min. A linear standard curve was obtained using values measured from standard samples of gradient concentrations. The measured sample values were then substituted into the function of the standard curve to calculate their HAT activity. Sample HAT activity =*B*×D/(Δ*TV*), where *B* is CoA amount from the standard curve (pmol), *T* is reaction time (min), *V* is the sample volume added into the reaction well (ml), and D is the dilution factor.

### Quantitative RT-PCR analysis

Total mRNAs were extracted by using a Aurum Total RNA Mini Kit (Bio-Rad), and cDNA was synthesized using RevertAid First Strand cDNA Synthesis Kit (Thermo Fisher Scientific) according to the manufacturer's instructions. Quantitative RT-PCR was performed using the Forget-Me-Not EvaGreen qPCR Master Mix with Rox (Biotium, Fremont, CA) and CFX96 Real-Time PCR Detection System (Bio-Rad). The sequences of all the primers were obtained from the National Center for Biotechnology Information database and listed in Table S1. For data analysis, the expressions of all genes were normalized using the ΔΔ cycle threshold method against human glyceraldehyde 3-phosphate dehydrogenase (GAPDH).

### Statistical analysis

Data were presented as mean±s.e.m. if not stated otherwise. Statistical analyses were conducted using GraphPad Prism (version 8.0.1). Two-tailed unpaired Student's *t*-test, or one-way or two way analysis of variance (ANOVA) with Bonferroni post-hoc test was used for the statistics among two or more conditions. The post hoc Tukey or Bonferroni test was adopted in the ANOVA analysis for the comparisons with equal or unequal sample sizes, respectively. *P*<0.05 was regarded as significant difference in tests of statistical inference. The statistical analysis was conducted using the log-rank (Mantel–Cox) test and hazard ratio (HR; 95% confidence intervals).

## Supplementary Material

10.1242/joces.259586_sup1 Supplementary information
