# Correction: Fluid shear stress regulates the survival of circulating tumor cells via nuclear expansion

**DOI:** 10.1242/jcs.261030

**Published:** 2023-02-28

**Authors:** Zichen Xu, Keming Li, Ying Xin, Kai Tang, Mo Yang, Guixue Wang, Youhua Tan

There was an error in *J. Cell Sci.* (2022) **135**, jcs259586 (doi:10.1242/jcs.259586).

The name of the author Kai Tang was spelled incorrectly (Kai Tan). The correct name appears above, and both the online full-text and PDF versions of the article have been updated. We apologise to the authors and readers for this error.

