# Supplementary material for: Fluid shear stress regulates the survival of circulating tumor cells via nuclear expansion
Source: J Cell Sci. 2022 May 26;135(10):jcs259586. doi: 10.1242/jcs.259586 (PMC10216746; doi:10.1242/jcs.259586)
Supplement: Supplementary information [file joces-135-259586-s1.pdf]

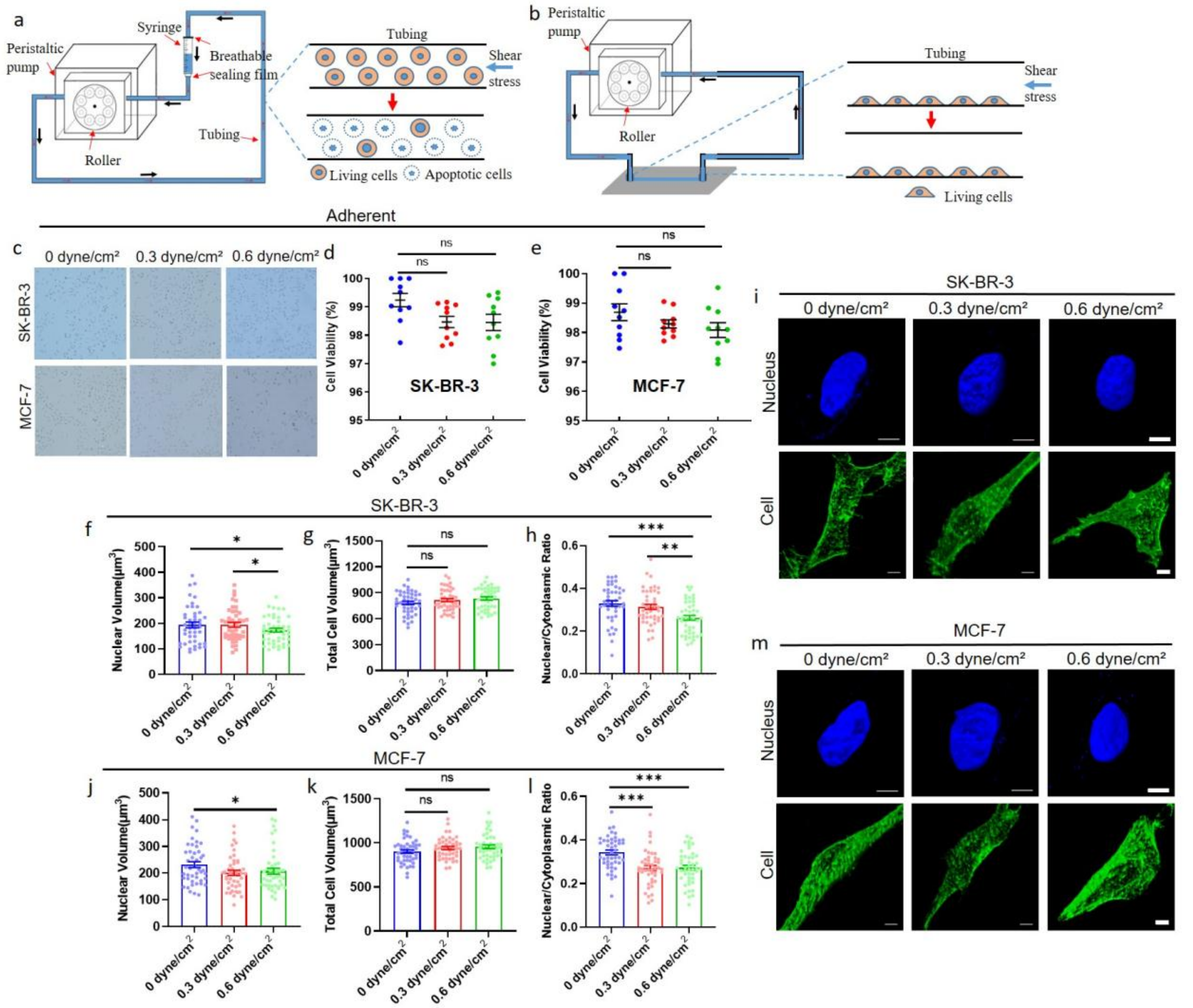

Fig. S1. Fluid shear stress does not influence the viability of adherent tumor cells while slightly reduces their nuclear size. (a) The circulatory system for suspended tumor cells. The system was composed of a peristatic pump, a silicone tubing, and a syringe as the cell solution reservoir. Tumor cells in suspension are subject to various levels of fluid shear stress. (b) The circulatory system for adherent tumor cells. The system was composed of a peristatic pump, a silicone tubing, and a μ-Slide I0.6 flow chamber chip. Tumor cells are attached to the collagen 1-coated chips and subjected to various levels of fluid shear stress. (c-e) Fluid shear stress has no significant effect on the viability of adherent tumor cells. SK-BR-3 and MCF-7 cells were attached on the surface of the flow chamber chip and subject to 0, 0.3 and 0.6 dyne/cm^2 shear stress for 12 h. The treated cells were then stained with PI and the percentage of PI negative cells was measured. The representative PI staining images of adherent tumor cells after the treatment of various shear stresses were shown in (c) and the viability was quantified in (d, e). Scale bar: 50 μm. n=10. (f-m) Fluid shear stress slightly

Journal of Cell Science • Supplementary information

decreases the nuclear but not cell size of adherent tumor cells. Breast cancer cells were treated similarly as in (c-e) and their cytoskeleton and nuclei were stained for the measurement of cell (g, k) and nuclear size (f, j) by confocal imaging. The representative cell and nuclear images of the treated tumor cells were shown in (i, m). Scale bar: 5 µm. n>45 cells. *, $p<0.05$; **, $p<0.01$; ***, $p<0.001$. ns: no significance.

Journal of Cell Science • Supplementary information

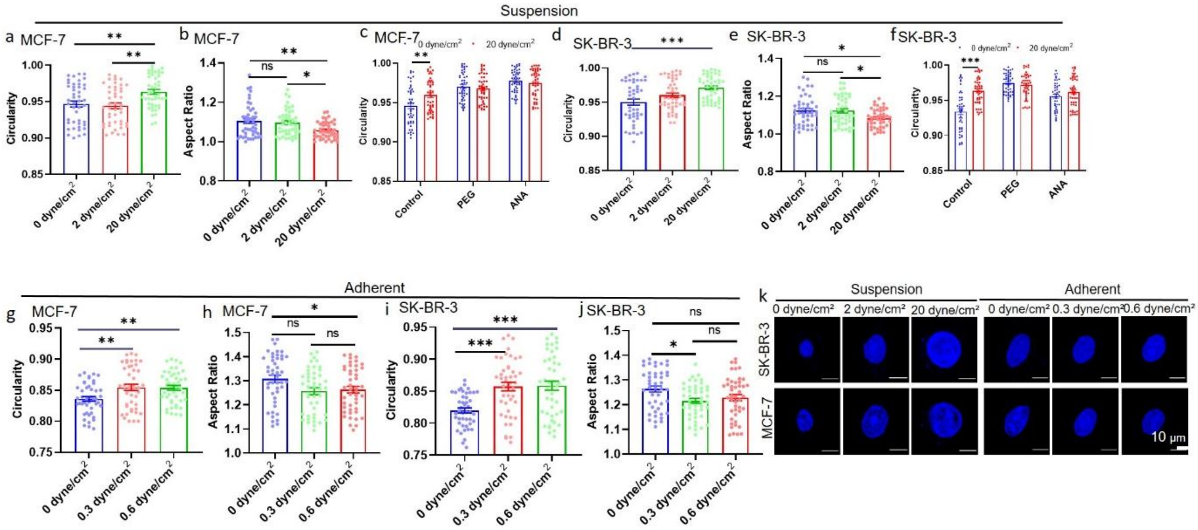

Fig. S2. Fluid shear stress increases the nuclear circularity and decreases the aspect ratio of both adherent and suspended tumor cells. (a, d) Fluid shear stress enhances the nuclear circularity of suspended tumor cells. (b, e) Fluid shear stress decreases the aspect ratio of suspended tumor cells. Suspended MCF-7 (a, b) and SK-BR-3 cells (d, e) were circulated under 0, 2, and 20 dyne/cm^2 shear stress for 12 h. The nuclei of these treated cells were stained with DAPI and their circularity and aspect ratio were quantified. n>45 cells. (c, f) Inhibiting HAT activity diminishes shear-induced increase in nuclear circularity. Breast cancer cells in suspension were treated similarly as in Fig.3i and j. The nuclear circularity of the treated cells were measured. n>45 cells. (g-j) Fluid shear stress enhances the nuclear circularity and reduces the aspect ratio of adherent tumor cells. Adherent MCF-7 (g, h) and SK-BR-3 cells (i, j) were treated under 0, 0.3, and 0.6 dyne/cm^2 shear stress for 12 h, respectively. The nuclei of these treated cells were stained with DAPI and their circularity and aspect ratio were quantified. n>45 cells. (k) Representative images of the nuclei in (a, d, g-j). Scale bar: 10 μm. *, p<0.05; **, p<0.01; ***, p<0.001. ns: no significance.

Journal of Cell Science • Supplementary information

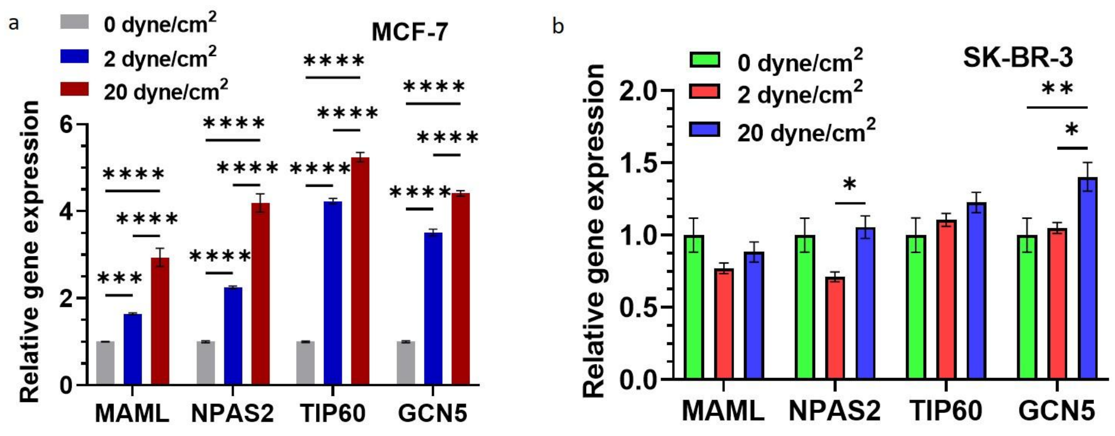

Fig. S3. Fluid shear stress up-regulates the expressions of HATs in suspended tumor cells. MCF-7 cells (a) and SK-BR-3 (b) in suspension were circulated under 0, 2, and 20 dyne/cm^2 shear stress for 12 h, respectively. The expressions of HATs in the treated cells were measured by quantitative RT-PCR, where ACTIN was used as a housekeeping gene. n=3. *, p<0.05; **, p<0.01; ***, p<0.001; ****, p<0.0001.

Journal of Cell Science • Supplementary information

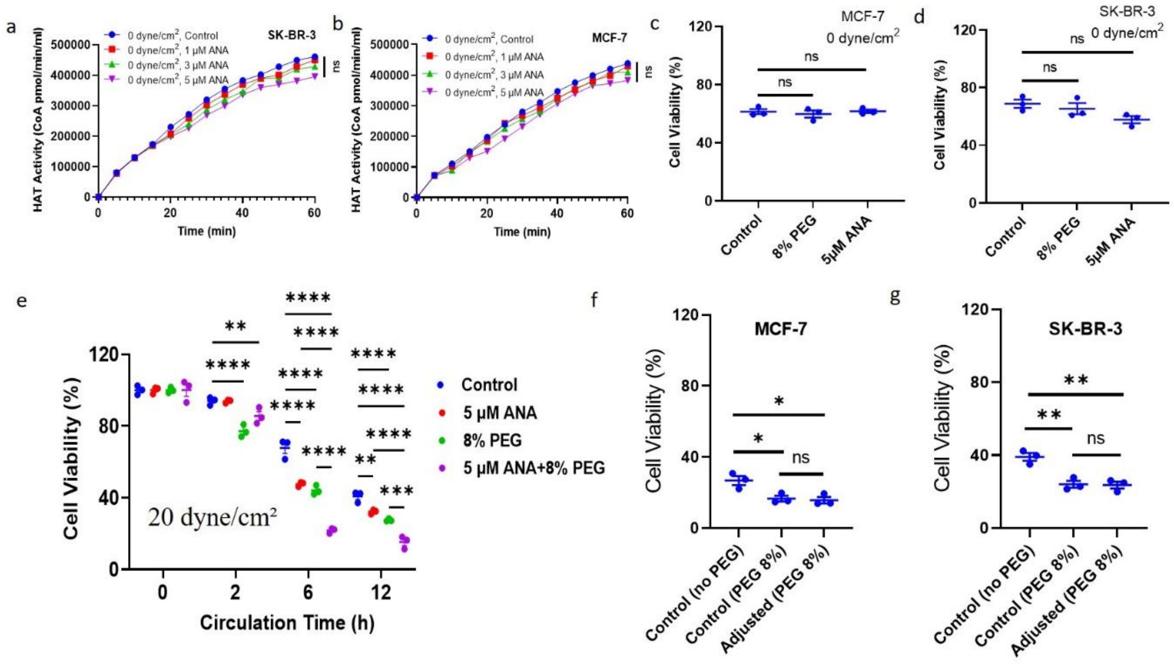

Fig. S4. The influence of PEG and HAT on HAT activity and the viability of suspended tumor cells. (a, b) Inhibiting HAT has no significant effect on the HAT activity of suspended tumor cells without shear stress treatment. SK-BR-3 (a) and MCF-7 cells (b) in suspension were treated under 0 dyne/cm^2 shear stress in the presence and absence of 1, 3, and 5 µM HAT inhibitor ANA, respectively. Their HAT activity was measured. n=3. (c, d) PEG and ANA have no significant effect on the viability of suspended tumor cells without shear stress treatment. MCF-7 (c) and SK-BR-3 cells (d) in suspension were treated under 0 dyne/cm^2 shear stress in the presence and absence of 8% PEG and 5 µM HAT inhibitor ANA for 12 h, respectively, when cell viability was measured by MTS assay. n=3. (e) The combination of PEG and ANA synergistically reduces the survival of suspended tumor cells under fluid shear stress. Suspended tumor cells were treated under 20 dyne/cm^2 shear stress in the presence of PEG, ANA, and PEG and ANA, respectively. Cell viability was measured by MTS assay. n=3. (f, g) PEG decreases tumor cell survival not through the effect on medium viscosity. Control no PEG: suspended tumor cells were subjected to shear flow with the flow rate of

Journal of Cell Science • Supplementary information

1.4718 ml/min in the medium without PEG; Control PEG 8%: suspended tumor cells were subjected to shear flow with the flow rate of 1.4718 ml/min in the medium with PEG; Adjusted PEG 8%: suspended tumor cells were subjected to shear flow with the flow rate of 1.376 ml/min in the medium with PEG. The viability of the treated cells was measured by MTS assay. n=3. *, p<0.05; **, p<0.01; ***, p<0.001; ****, p<0.0001. ns: no significance.

Journal of Cell Science • Supplementary information

Table S1. The primer sequence of all the genes used for quantitative RT-PCR

Genes		Quantitative RT-PCR
GAPDH	5’ primer	TGAAGGTCGGAGTCAACGGATTTGG
	3’ primer	GGAGGCCATGTGGGCCATGAG
MAML1	5’ primer	CCCCAGTGAGTCATTTCCTCT
	3’ primer	GAGGTTGCTTTGCGATATGGA
NPAS2	5’ primer	TCTGGATCACAGAGCACCTC
	3’ primer	CAGGAGCTCCAGGTCATCA
TIP60	5’ primer	CAGGACAGCTCTGATGGAATAC
	3’ primer	AGAGGACAGGCAATGTGGTGAG
GCN5	5’ primer	GTGCTGTCACCTCGAATGAG
	3’ primer	TGGAGAAACCCTGCTTTTTGA
Bad	5’ primer	CCTCAGGCCTATGCAAAAAG
	3’ primer	AAACCCAAAACTTCCGATGG
Bax	5’ primer	GAGCAGCCGCCCCAGGATG
	3’ primer	GGTGAGCGAGGCGGTGAGGAC
p53	5’ primer	CCCGAGTATCTGGAAGACAG
	3’ primer	ATAGGTCGGCGGTTCAT
p73	5’ primer	GGACGTAGCGAAATCGGGGTTC
	3’ primer	ACCCCGAACATCGACGTCCG
SOD2	5’ primer	ACGTTGGATGCTGTGCTTTCTCGTCTTCAG
	3’ primer	ACGTTGGATGTTCTGCCTGGAGCCCAGATAC
PUMA	5’ primer	GCGAGACTGTGGCCTTGTGT
	3’ primer	CGTTCCAGGGTCCACAAAGT
Caspase3	5’ primer	ATGGGAGCAAGTCAGTGGAC
	3’ primer	TTGAGGTAGCTGCACTGTGG
Bcl2	5’ primer	CCCACATACACGGCTAGAAAAGG
	3’ primer	CCATGAAAACAAGGGCTGGAAAA
Bcl-XL	5’ primer	GGGCATTCAGTGACCTGACA
	3’ primer	GCATTGTTCCCATAGAGTTC
Twist1	5’ primer	TTCTCGGTCTGGAGGATGGA
	3’ primer	CCCACGCCCTGTTTC
Actin	5’ primer	CACCATTGGCAATGAGCGGTTC
	3’ primer	AGGTCTTTGCGGATGTCCACGT

Journal of Cell Science • Supplementary information
