# Supplementary material for: Fluid shear stress regulates the survival of circulating tumor cells via nuclear expansion
Source: J Cell Sci. 2022 May 26;135(10):jcs259586. doi: 10.1242/jcs.259586 (PMC10216746; doi:10.1242/jcs.259586)
Supplement: Supplementary file 1 [file joces-135-259586_review_history.pdf]

Fluid shear stress regulates the survival of circulating tumor cells via nuclear expansion

Zichen Xu, Keming Li, Ying Xin, Kai Tang, Mo Yang, Guixue Wang and Youhua Tan
DOI: 10.1242/jcs.259586

Editor: Andrew Ewald

Review timeline

Original submission:	12 November 2021
Editorial decision:	7 January 2022
First revision received:	16 March 2022
Editorial decision:	19 April 2022
Second revision received:	20 April 2022
Editorial decision:	27 April 2022
Third revision received:	27 April 2022
Accepted:	27 April 2022

Original submission

First decision letter

MS ID#: JOCES/2021/259586

MS TITLE: Fluid shear stress regulates the survival of suspended circulating tumor cells via histone acetylation-mediated nuclear expansion

AUTHORS: Zichen Xu, Keming Li, Ying Xin, Kai Tang, Mo Yang, Guixue Wang, and Youhua Tan
ARTICLE TYPE: Research Article

We have now reached a decision on the above manuscript.

To see the reviewers' reports and a copy of this decision letter, please go to: https://submit-jcs.biologists.org and click on the 'Manuscripts with Decisions' queue in the Author Area. (Corresponding author only has access to reviews.)

As you will see, the reviewers share enthusiasm for the study but raise a number of substantial criticisms that prevent me from accepting the paper at this stage. These include aspects of assay design, normalization, control of variables, and whether there are confounding influences on the ability to interpret which effects arise from cell response to shear stress. They suggest, however, that a revised version might prove acceptable, if you can address their concerns. If you think that you can deal satisfactorily with the criticisms on revision, I would be pleased to see a revised manuscript. We would then return it to the reviewers.

We are aware that you may be experiencing disruption to the normal running of your lab that makes experimental revisions challenging. If it would be helpful, we encourage you to contact us to discuss your revision in greater detail. Please send us a point-by-point response indicating where you are able to address concerns raised (either experimentally or by changes to the text) and where you will not be able to do so within the normal timeframe of a revision. We will then provide further guidance. Please also note that we are happy to extend revision timeframes as necessary.

© 2022. Published by The Company of Biologists under the terms of the Creative Commons Attribution License (https://creativecommons.org/licenses/by/4.0/).

Please ensure that you clearly highlight all changes made in the revised manuscript. Please avoid using 'Tracked changes' in Word files as these are lost in PDF conversion.
I should be grateful if you would also provide a point-by-point response detailing how you have dealt with the points raised by the reviewers in the 'Response to Reviewers' box. Please attend to all of the reviewers' comments. If you do not agree with any of their criticisms or suggestions please explain clearly why this is so.

Reviewer 1

Advance summary and potential significance to field

CTC's are important to understand and potentially possess uniquely druggable pathways. This submission suggests nuclear and/or cell size increase with shear due to HATs, with drugs affecting viability under shear.

Comments for the author

CTC's are important to understand and potentially possess uniquely druggable pathways. This submission suggests nuclear and/or cell size increase with shear due to HATs, with drugs affecting viability under shear.
These in vitro studies of breast cancer lines under shear have a number intriguing result, but a number of concerns temper enthusiasm.
1. Fig.1a,b indicates many or most cells exposed to high shear for hour are not viable based on an MTS assay, but the assay seems to be delayed (by 12 h) and even involve cell settling and attachment "100 µL of cell suspension was collected from the circulatory system and then added into 1 well of a 96-well plate. After 12 h of incubation, 20 µL of sterilized CellTiter Aqueous One Solution (5 mg/mL; Promega) was added to each well, and the plate was incubated at 37°C for 4 h." In contrast, Fig.1c-j, Fig.2, etc. do not involve a 12 h delay or attachment. The authors need to measure viability kinetics more carefully to rule out the possibility that nuclear size changes, etc reflect early stages of shear induced death.
2. Addition of PEG in Fig.2 is a good idea, but PEG affects viscosity. Also since PEG shrinks cells and nuclei per Fig.2, the fluid shear stress is suppressed. The authors need to discuss how they factored these effects into their analyses.
3. If ANA and PEG are in the same shear response pathway, then their combination under shear should have no added effect. Is this true?
4. PCR results (Fig.2b,f, Fig.3) need housekeeping genes, preferrably more than one. Also, MCF7 cells don't seem to show overall increases in HAT levels in Fig.2f at intermediate shear: one gene is up, and two seem down. If Fig.2b,f are the specific HAT's inhibited by ANA, then knockdowns should have similar effects as ANA under shear.
5. Survival analysis is good to see, but Fig.4 seems to be data for primary tumor which is '0 shear' and thus a condition in vitro that does not affect the HAT's.
Furthermore, other public datasets do not provide significance and should be discussed:
(A) "MAML1 is not prognostic in breast cancer"
https://www.proteinatlas.org/ENSG00000161021-MAML1/pathology/breast+cancer
(B) "NPAS2 is not prognostic in breast cancer"
https://www.proteinatlas.org/ENSG00000170485-NPAS2/pathology/breast+cancer
(C) "KAT5 is not prognostic in breast cancer"
https://www.proteinatlas.org/ENSG00000172977-KAT5/pathology/breast+cancer

Reviewer 2

Advance summary and potential significance to field

The authors address an important problem, the response of breast cancer cells to the mechanical stimulus of fluid shear stress. These behaviors can have important implications for bloodborne/distant metastasis. It is recommended to perform some additional quantitative analysis of the nuclear image data, and to place this work in the context of recent findings related to Piezo1 in cancer apoptosis. My specific suggestions are listed in the next text field.

© 2022. Published by The Company of Biologists under the terms of the Creative Commons Attribution License (https://creativecommons.org/licenses/by/4.0/).

Comments for the author

1. blood shear flow induced substantial apoptosis of CTCs in a stress-dependent manner: authors should discuss the role of Piezo1 in this process. Reported role of Piezo1 in paper by Hope et al. in Cell Death and Disease 2019. You mention the role of Piezo1 in shear flow nuclear shrinkage on line 104-5 but strangely do not discuss its role in apoptosis of cancer cells under flow.
2. Authors should be more precise in reporting the estimated shear stress magnitude. The shear stress value calculated using Poiseuille's Law (line 135) is actually the WALL shear stress, shear stress varies linearly with radial position, with maximum at the wall and zero in the center. So only cells that are near the wall will experience a shear stress magnitude close to the reported value.
3. A high throughput single cell measure of cell viability such as flow cytometry would greatly enhance the bulk measurements obtained via MTS assay.
4. Reports of nuclear size are interesting, but it would also be interesting to see any change in nuclear SHAPE, as quantified by aspect ratio, shape factor, etc. This is a missed opportunity.

First revision

Author response to reviewers' comments

Reviewer 1 Advance summary and potential significance to field
CTC's are important to understand and potentially possess uniquely druggable pathways. This submission suggests nuclear and/or cell size increase with shear due to HATs, with drugs affecting viability under shear.

Reviewer 1 Comments for the author
CTC's are important to understand and potentially possess uniquely druggable pathways. This submission suggests nuclear and/or cell size increase with shear due to HATs, with drugs affecting viability under shear.
These in vitro studies of breast cancer lines under shear have a number intriguing result, but a number of concerns temper enthusiasm.

1. Fig.1a,b indicates many or most cells exposed to high shear for hour are not viable based on an MTS assay, but the assay seems to be delayed (by 12 h) and even involve cell settling and attachment "100 µL of cell suspension was collected from the circulatory system and then added into 1 well of a 96-well plate. After 12 h of incubation, 20 µL of sterilized CellTiter Aqueous One Solution (5 mg/mL; Promega) was added to each well, and the plate was incubated at 37°C for 4 h." In contrast, Fig.1c-j, Fig.2, etc. do not involve a 12 h delay or attachment. The authors need to measure viability kinetics more carefully to rule out the possibility that nuclear size changes, etc reflect early stages of shear induced death.
Response: Thanks for the comment. Following this reviewer's suggestion, we conducted additional experiments and measured cell viability using Calcein-acetoxymethyl (AM) and propidium iodide (PI) staining and Annexin V assay through flow cytometry. Breast tumor cells were collected immediately after the treatment with 0, 2, 20 dyne/cm2 shear flow for Calcein-AM/PI staining or Annexin V assay. The new data in Fig. 1c-f show that the viability of suspended tumor cells decreases as the magnitude of shear stress increases, which is consistent with the results measured by MTS. Hence, all these results suggest the suppressive effect of fluid shear stress on the viability of suspended tumor cells. We have added the relevant description about the new results in the revised manuscript (see Line 128-129 at page 6).

2. Addition of PEG in Fig.2 is a good idea, but PEG affects viscosity. Also, since PEG shrinks cells and nuclei per Fig.2, the fluid shear stress is suppressed. The authors need to discuss how they factored these effects into their analyses.
Response: Thanks for the comments. The addition of PEG could change the medium viscosity, consequently affecting fluid shear stress based on the Poiseuille's law. According to the Poiseuille's law and Newton's law for internal friction in fluid, 8% PEG increases the medium viscosity by 6.5%. To take the altered viscosity into account and maintain the same magnitude of fluid shear stress (20 dyne/cm2 in the presence of 8% PEG), we reduced the shear rate from 1.4718 ml/min for

© 2022. Published by The Company of Biologists under the terms of the Creative Commons Attribution License (https://creativecommons.org/licenses/by/4.0/).

control medium to 1.376 ml/min for PEG medium. The new results in Fig. S5f and S5g show that shear flow with the flow rate of 1.376 ml/min (Adjusted PEG 8%) and 1.4718 ml/min (Control PEG 8%) decreases the viability of suspended tumor cells in the PEG medium compared to shear flow with the flow rate of 1.4718 ml/min in the medium without PEG (Control no PEG). There is no difference in cell viability between different flow rate (1.4718 ml/min and 1.376 ml/min) when considering the potential effect of PEG on medium viscosity. These new results suggest that the addition of PEG slightly increases the medium viscosity while does not significantly impact cell viability via the effect on viscosity.
The addition of PEG reduces both sizes of the whole cell and nucleus under 20 dyne/cm2 shear stress as well as the ratio of nuclear/cytoplasmic size (Fig. 3i and j), suggesting that PEG preferentially suppresses the nuclear size. Nevertheless, to explore the influence of nuclear size, it is ideal to specifically inhibit the nuclear but not cell size under the treatment of fluid shear stress. We have added the new results (Fig. S5) and the relevant discussion in the revised manuscript (see Line 189-191 at page 8 and Line 213-219 at page 9).

3. If ANA and PEG are in the same shear response pathway, then their combination under shear should have no added effect. Is this true?
Response: Thanks for the comment. Our results show that both ANA and PEG reduce the nuclear volume and viability of suspended tumor cells through the inhibition of HAT activity under fluid shear stress. Following this reviewer's suggestion, we tested the combined influence of ANA and PEG on cell viability and found that the combination of ANA and PEG further reduced the viability of suspended tumor cells under fluid shear stress compared to the effect of a single factor (either ANA or PEG; new results Fig. S5e). The combination of ANA and PEG may have synergistic inhibitory effect on HAT activity and nuclear volume, thus impairing the viability of suspended tumor cells under fluid shear stress. We have added the new results (Fig. S5e) and the relevant description in the revised manuscript (see Line 209-212 at page 9).

4. PCR results (Fig.2b,f, Fig.3) need housekeeping genes, preferably more than one. Also, MCF7 cells don't seem to show overall increases in HAT levels in Fig.2f at intermediate shear: one gene is up, and two seem down. If Fig.2b,f are the specific HAT's inhibited by ANA, then knockdowns should have similar effects as ANA under shear.
Response: Thanks for the comments. Following the suggestion, we repeated these q-PCR experiments based on another housekeeping gene, ACTIN, and obtained similar results (with GAPDH as the housekeeping gene) as shown in Fig S4. Therefore, our previous and new results suggest that fluid shear stress may regulate the survival of suspended tumor cells possibly via the effect on histone acetylation. However, it remains unclear which HATs play the major role in this process. In the future, it is worthy to identify the major HATs (via specific knockdown using siRNAs) that regulate shear-induced histone acetylation in suspended tumor cells. We have added the relevant discussion in the revised manuscript (see Line 167-168 at page 8 and Line 178-180 at page 8).

5. Survival analysis is good to see, but Fig.4 seems to be data for primary tumor which is '0 shear' and thus a condition in vitro that does not affect the HAT's. Furthermore, other public datasets do not provide significance and should be discussed:
(A) "MAML1 is not prognostic in breast cancer"
https://www.proteinatlas.org/ENSG00000161021-MAML1/pathology/breast+cancer
(B) "NPAS2 is not prognostic in breast cancer"
https://www.proteinatlas.org/ENSG00000170485-NPAS2/pathology/breast+cancer
(C) "KAT5 is not prognostic in breast cancer"
https://www.proteinatlas.org/ENSG00000172977-KAT5/pathology/breast+cancer

Response: Thanks for the comments. We agree with this reviewer that the public datasets of primary CTCs from breast cancer patients should be utilized for survival analysis, since only a small subpopulation of primary tumor cells have the ability to enter into the vasculature and eventually generate distant metastases. Thus, the gene expression pattern of CTCs could be very different and unique compared to that of primary tumor cells. However, the available CTC datasets are limited, which challenges the analysis of the correlation between HAT-related genes of CTCs and patient survival. In addition, the inter-tumoral heterogeneity is also a hallmarker of primary tumors and represents distinct genetic and phenotypic patterns from patients to patients. Therefore, it is possible to observe different relationships between the expressions of certain genes and patient

© 2022. Published by The Company of Biologists under the terms of the Creative Commons Attribution License (https://creativecommons.org/licenses/by/4.0/).

survival among different datasets. This could be one limitation of this study, which has been discussed in the revised manuscript (see Line 328-334 at page 14).
Nevertheless, our in vitro studies show that HAT activity can facilitate the survival of suspended circulating tumor cells in the circulation system, suggesting that tumor cell subpopulations with high HAT activity in the primary tumor may better survive throughout blood circulation and eventually generate metastatic tumors, thereby limiting patient survival.

Reviewer 2 Advance summary and potential significance to field
The authors address an important problem, the response of breast cancer cells to the mechanical stimulus of fluid shear stress. These behaviors can have important implications for bloodborne/distant metastasis. It is recommended to perform some additional quantitative analysis of the nuclear image data, and to place this work in the context of recent findings related to Piezo1 in cancer apoptosis. My specific suggestions are listed in the next text field.

Reviewer 2 Comments for the author
1. blood shear flow induced substantial apoptosis of CTCs in a stress-dependent manner: authors should discuss the role of Piezo1 in this process. Reported role of Piezo1 in paper by Hope et al. in Cell Death and Disease 2019. You mention the role of Piezo1 in shear flow nuclear shrinkage on line 104-5 but strangely do not discuss its role in apoptosis of cancer cells under flow.
Response: Thanks for the comments. Our results have demonstrated that tumor cells in suspension respond to fluid shear stress in blood circulation through histone acetylation-mediated nuclear expansion, which protects CTCs from shear-induced elimination. These findings provide insight into the mechanism by which suspended cells sense and respond to mechanical cues. However, the mechanotransduction mechanism of suspended tumor cells in response to fluid shear stress remains unclear. We agree with this reviewer that Piezo1 may play an important role in tumor cell mechanosensing. Following the suggestion, we have added the following discussion about the significance of Piezo1 in the revised manuscript (see Line 307-320 at page 13 and 14).
“We have demonstrated that suspended CTCs can sense and respond to fluid shear stress in the vasculature through histone acetylation-mediated nuclear expansion, which protects them from shear-induced elimination. However, the mechanotransduction mechanism has not been completely understood, especially the mechanosensor by which suspended cells sense fluid shear stress. It is well known that adherent tumor cells can sense mechanical cues through Piezo1 (Dombroski et al., 2021). For example, glioblastoma cells sense matrix stiffness via Piezo1-mediated mechanotransduction (Chen et al., 2018). Fluid shear stress induces nuclear shrinkage in adherent epithelial cells through Piezo1-mediated calcium influx (Jetta et al., 2019). Recent evidence shows that fluid shear stress enhances the sensitivity of suspended tumor cells to TRAIL-mediated apoptosis via Piezo1-mediated calcium influx and activation of calpains (Hope et al., 2019). Therefore, it is possible that suspended tumor cells may sense fluid shear stress via Piezo1 channel, which may further affect histone acetylation and nuclear size. In the future, it is important to elucidate the role of Piezo1 in the mechanosensing of suspended cells.”

2. Authors should be more precise in reporting the estimated shear stress magnitude. The shear stress value calculated using Poiseuille’s Law (line 135) is actually the WALL shear stress, shear stress varies linearly with radial position, with maximum at the wall and zero in the center. So only cells that are near the wall will experience a shear stress magnitude close to the reported value.
Response: Thanks for the comment. We agree with the reviewer that the reported shear stress in this study represents wall shear stress on the tube wall and changes radially within the tube. To clarify this issue, we have clearly explained this in the revised manuscript (see Line 124-128 at page 6).

3. A high throughput single cell measure of cell viability such as flow cytometry would greatly enhance the bulk measurements obtained via MTS assay.

Response: Thanks for the comment. Following this reviewer’s suggestion, we measured the viability of suspended tumor cells after shear treatment by both Calcein-AM/PI staining or Annexin V assay through flow cytometry. The new results in Fig. 1 show that the viability of suspended tumor cells decreases as the magnitude of shear stress increases, which is consistent with the results measured by MTS assay. Hence, all these results demonstrate that fluid shear stress affects the viability of suspended tumor cells in a magnitude dependent manner. We have added the relevant description in the revised manuscript (see Line 128-129 at page 6).

© 2022. Published by The Company of Biologists under the terms of the Creative Commons Attribution License (https://creativecommons.org/licenses/by/4.0/).

4. Reports of nuclear size are interesting, but it would also be interesting to see any change in nuclear SHAPE, as quantified by aspect ratio, shape factor, etc. This is a missed opportunity.
Response: Thanks for the suggestion. We have analyzed the circularity of tumor cell nuclei after various shear treatments in Fig. S3. Following the suggestion, we further quantified the aspect ratio of nuclei. The results show that fluid shear stress reduces the aspect ratio of the nuclei in suspended breast cancer cells in a magnitude dependent manner (Fig. S3b, e, h, and j). We have added the relevant description in the revised manuscript (see Line 147-149 at page 7).

Reference:
Chen, X., Wanggou, S., Bodalia, A., Zhu, M., Dong, W., Fan, J. J., Yin, W. C., Min, H.-K., Hu, M., Draghici, D., et al. (2018). A Feedforward Mechanism Mediated by Mechanosensitive Ion Channel PIEZO1 and Tissue Mechanics Promotes Glioma Aggression. Neuron 100, 799-815.e7.
Dombroski, J. A., Hope, J. M., Sarna, N. S. and King, M. R. (2021). Channeling the force: Piezo1 mechanotransduction in cancer metastasis. Cells 10, 2815.
Hope, J. M., Lopez-Cavestany, M., Wang, W., Reinhart-King, C. A. and King, M. R. (2019). Activation of Piezo1 sensitizes cells to TRAIL-mediated apoptosis through mitochondrial outer membrane permeability. Cell Death Dis. 10, 837.
Jetta, D., Gottlieb, P. A., Verma, D., Sachs, F. and Hua, S. Z. (2019). Shear stress-induced nuclear shrinkage through activation of Piezo1 channels in epithelial cells. J. Cell Sci. 132, jcs226076.

Second decision letter

MS ID#: JOCES/2021/259586

MS TITLE: Fluid shear stress regulates the survival of circulating tumor cells via histone acetylation-mediated nuclear expansion

AUTHORS: Zichen Xu, Keming Li, Ying Xin, Kai Tang, Mo Yang, Guixue Wang, and Youhua Tan
ARTICLE TYPE: Research Article

We have now reached a decision on the above manuscript.

To see the reviewers' reports and a copy of this decision letter, please go to: https://submit-jcs.biologists.org and click on the 'Manuscripts with Decisions' queue in the Author Area. (Corresponding author only has access to reviews.)

As you will see, the reviewers found the revised manuscript to be significantly responsive to their concerns. However, Reviewer 1 has identified some remaining issues that need to be addressed through some combination of data re-analysis and revisions to the figures and manuscripts. It is also important the claims made in the text are fully supported in the figures; Reviewer 1's concerns partly result from a mismatch in this area. I hope that you will be able to carry these out because I would like to be able to accept your paper, depending on further comments from reviewers.

Please ensure that you clearly highlight all changes made in the revised manuscript. Please avoid using 'Tracked changes' in Word files as these are lost in PDF conversion.

I should be grateful if you would also provide a point-by-point response detailing how you have dealt with the points raised by the reviewers in the 'Response to Reviewers' box. Please attend to all of the reviewers' comments. If you do not agree with any of their criticisms or suggestions please explain clearly why this is so.

Reviewer 1

Advance summary and potential significance to field

see previous

© 2022. Published by The Company of Biologists under the terms of the Creative Commons Attribution License (https://creativecommons.org/licenses/by/4.0/).

Comments for the author

The authors were reasonably responsive with their revision, but several issues require further attention.
1. They report "the combination of ANA and PEG further reduced the viability of suspended tumor cells under fluid shear stress compared to the effect of a single factor". If they know or show that each factor is used in the linear response regime and also well below saturation, then do they have synergy (which exceeds simple additivity)? Or do they have an effect that is less than additive? The latter could hint at being in the same pathway.
2. Given the fact that they only have one modestly specific pharmacological perturbation of the postulated molecular pathway, and that they understand more can be done, I find it unconvincing for them to point out "In the future, it is worthy to identify the major HATs (via specific knockdown using siRNAs) that regulate shear-induced histone acetylation in suspended tumor cells." They simply need to do the knockdowns, and maybe +/-ANA to be convincing of the claims.
3. The authors continue to show the same publicly available patient survival data even though they recognize other public data differs. It is misleading to only discuss other data; they need to replace the current Figure with a proper summary (such as a Table) that indicates what can be seen in two or more datasets in the public domain.

Reviewer 2

Advance summary and potential significance to field

The authors have done a good job of addressing my comments on the original submission.
In my opinion this manuscript is acceptable for publication in JCS and is a nice study.

Comments for the author

None.

Second revision

Author response to reviewers' comments

Reviewer 1 Comments for the author
The authors were reasonably responsive with their revision, but several issues require further attention.

1. They report "the combination of ANA and PEG further reduced the viability of suspended tumor cells under fluid shear stress compared to the effect of a single factor". If they know or show that each factor is used in the linear response regime and also well below saturation, then do they have synergy (which exceeds simple additivity)? Or do they have an effect that is less than additive? The latter could hint at being in the same pathway.

Response: Thanks for the comment. We agree with this reviewer that the current results cannot suggest the synergistic effect of ANA and PEG on the survival of suspended tumor cells. To clarify this issue, we revised the following statement in the revised manuscript (see Line 206-207 at page 9). “In addition, the combination of PEG and ANA further reduced cell viability to an even lower level”

2. Given the fact that they only have one modestly specific pharmacological perturbation of the postulated molecular pathway, and that they understand more can be done, I find it unconvincing for them to point out "In the future, it is worthy to identify the major HATs (via specific knockdown using siRNAs) that regulate shear-induced histone acetylation in suspended tumor cells." They simply need to do the knockdowns, and maybe +/-ANA to be convincing of the claims.

© 2022. Published by The Company of Biologists under the terms of the Creative Commons Attribution License (https://creativecommons.org/licenses/by/4.0/).

Response: Thanks for the comment. The acetylation of lysine residues is reversibly catalyzed by histone acetyltransferase (HATs) and histone deacetylases (HDACs), which are responsible for adding and removing acetyl groups from the N-terminal tail of the histone, respectively (Wu et al., Front Oncol. 2020 Nov 11;10:560487). Therefore, the balance between HATs and HDACs largely determines the histone acetylation level. The mechanism underlying shear-induced histone acetylation may be far more complexed than the effect of HATs. Nevertheless, our findings show that fluid shear stress up-regulates the expressions of HATs, which may partially explain the shear-induced increase in the histone acetylation level. To clarify this issue, we deleted the following statement in the revised manuscript.
“However, it remains unclear which HATs play the major role in shear-induced histone acetylation of suspended tumor cells, which will be explored in the future.”
Further, we agree with this reviewer that comprehensively elucidating the mechanism underlying shear-induced histone acetylation and nuclear expansion is critical. In the future, the RNA sequencing technique will be utilized to identify the potential targets, including HATs, HDACs, and others. The roles of these targets in shear-induced histone acetylation and nuclear expansion will be further dissected. However, this is beyond the scope of this study.

3. The authors continue to show the same publicly available patient survival data even though they recognize other public data differs. It is misleading to only discuss other data; they need to replace the current Figure with a proper summary (such as a Table) that indicates what can be seen in two or more datasets in the public domain.

Response: Thanks for the comment. We agree with this reviewer that the public datasets of primary CTCs from breast cancer patients should be utilized for survival analysis, since only a small subpopulation of primary tumor cells have the ability to enter into the vasculature and eventually generate distant metastases. However, the available CTC datasets are limited, which challenges the analysis of the correlation between HAT-related genes of CTCs and patient survival. Further, our recent study and current findings have shown that the properties of suspended tumor cells (mimicking CTCs) that survive in the shear flow are distinct from the tumor cells that are attached to solid substrates (mimicking tumor cells in the primary tumor) (Xin et al., Biophys J . 2019 May 21;116(10):1803-1814). These findings are supported by many other studies that show the difference between primary tumor cells and CTCs (Pantel & Speicher, Oncogene (2016) 35, 1216-1224). A recent study reports that CTCs have many unique mutations that are not found in the primary tumors (Chang et al., Diagnostics (Basel). 2021 Jun; 11(6): 1102.). Therefore, the gene expression pattern of CTCs could be very different and unique compared to that of primary tumor cells. It thus may be not appropriate to utilize the data of primary tumor cells from any public datasets in the context of the current study in which tumor cells are in suspension. To avoid the potential misleading, we decided to remove Figure 5, which analyzed the relationship between patient survival and the expressions of specific genes in the primary tumor.

Reviewer 2 Advance summary and potential significance to field
The authors have done a good job of addressing my comments on the original submission. In my opinion this manuscript is acceptable for publication in JCS and is a nice study.

Reviewer 2 Comments for the author
None.

Response: We thank Reviewer 2’s efforts and constructive comments.

© 2022. Published by The Company of Biologists under the terms of the Creative Commons Attribution License (https://creativecommons.org/licenses/by/4.0/).

Third decision letter

MS ID#: JOCES/2021/259586

MS TITLE: Fluid shear stress regulates the survival of circulating tumor cells via histone acetylation-mediated nuclear expansion

AUTHORS: Zichen Xu, Keming Li, Ying Xin, Kai Tang, Mo Yang, Guixue Wang, and Youhua Tan
ARTICLE TYPE: Research Article

We have now reached a decision on the above manuscript.

To see the reviewers' reports and a copy of this decision letter, please go to: https://submit-jcs.biologists.org and click on the 'Manuscripts with Decisions' queue in the Author Area. (Corresponding author only has access to reviews.)

As you will see, the reviewer sees a substantial gap between the strength of the experimental conclusions and the strength of the language used in the title and abstract. I think it is essential to bring these into alignment. The reviewer suggests language for both. If you would like to propose edits, you may, but they must convey the essential points made by the reviewer. There is still unresolved ambiguity in the study and this needs to be accurately reflected in the title and abstract. I hope that you will be able to carry these out because I would like to be able to accept your paper.

Please ensure that you clearly highlight all changes made in the revised manuscript. Please avoid using 'Tracked changes' in Word files as these are lost in PDF conversion.

I should be grateful if you would also provide a point-by-point response detailing how you have dealt with the points raised by the reviewers in the 'Response to Reviewers' box. Please attend to all of the reviewers' comments. If you do not agree with any of their criticisms or suggestions please explain clearly why this is so.

Reviewer 1

Advance summary and potential significance to field

See previous and comments to authors.

Comments for the author

Given that the authors have only one drug suggestive of the claimed molecular mechanism, they must edit the title and abstract as following (or similar):
1. TITLE: "Fluid shear stress regulates the survival of circulating tumor cells via nuclear expansion"
2. ABSTRACT (edits in CAPS): "Distant metastasis ... SHEAR flow promotes histone acetylation in suspended tumor cells, AND RESULTS WITH ONE DRUG SHOW THAT inhibition shear-induced nuclear expansion IS SUPPRESSED, suggesting that shear stress MIGHT increase nuclear size through histone acetylation. Suppressing histone acetylation-mediated nuclear expansion enhances shear-induced apoptosis of CTCs. These findings SUGGEST that suspended tumor cells respond to shear stress through histone acetylation-mediated nuclear expansion, which protects CTCs from shear-induced destruction. Our study elucidates a unique mechanism underlying the mechanotransduction of suspended CTCs to shear flow, which may hold therapeutic promise for CTC eradication.

© 2022. Published by The Company of Biologists under the terms of the Creative Commons Attribution License (https://creativecommons.org/licenses/by/4.0/).

Third revision

Author response to reviewers' comments

Reviewer 1 Comments for the author
Given that the authors have only one drug suggestive of the claimed molecular mechanism, they must edit the title and abstract as following (or similar):
1. TITLE: "Fluid shear stress regulates the survival of circulating tumor cells via nuclear expansion"
2. ABSTRACT (edits in CAPS): "Distant metastasis ... SHEAR flow promotes histone acetylation in suspended tumor cells, AND RESULTS WITH ONE DRUG SHOW THAT inhibition shear-induced nuclear expansion IS SUPPRESSED, suggesting that shear stress MIGHT increase nuclear size through histone acetylation. Suppressing
histone acetylation-mediated nuclear expansion enhances shear-induced apoptosis of CTCs. These findings SUGGEST that suspended tumor cells respond to shear stress through histone acetylation-mediated nuclear expansion, which protects CTCs from shear-induced destruction. Our study elucidates a unique mechanism underlying the mechanotransduction of suspended CTCs to shear flow, which may hold therapeutic promise for CTC eradication.

Response: Thanks for the comment. We followed the suggestion and revised the title and abstract as follows.

Title: “Fluid shear stress regulates the survival of circulating tumor cells via nuclear expansion”

Abstract:
“Distant metastasis is mainly through hematogenous dissemination, where suspended circulating tumor cells (CTCs) experience considerable level of fluid shear stress. We recently reported that shear flow induced substantial apoptosis of CTCs, while a small subpopulation could still persist. However, how suspended tumor cells survive in shear flow remains poorly understood. This study finds that fluid shear stress eliminates the majority of suspended CTCs and increases nuclear size while has no effect on the viability of adherent tumor cells and decreases their nuclear size. Shear flow promotes histone acetylation in suspended tumor cells, the inhibition of which using one drug suppresses shear-induced nuclear expansion, suggesting that shear stress might increase nuclear size through histone acetylation. Suppressing histone acetylation-mediated nuclear expansion enhances shear-induced apoptosis of CTCs. These findings suggest that suspended tumor cells respond to shear stress through histone acetylation-mediated nuclear expansion, which protects CTCs from shear-induced destruction. Our study elucidates a unique mechanism underlying the mechanotransduction of suspended CTCs to shear flow, which may hold therapeutic promise for CTC eradication.”

Fourth decision letter

MS ID#: JOCES/2021/259586

MS TITLE: Fluid shear stress regulates the survival of circulating tumor cells via nuclear expansion

AUTHORS: Zichen Xu, Keming Li, Ying Xin, Kai Tang, Mo Yang, Guixue Wang, and Youhua Tan
ARTICLE TYPE: Research Article

I am happy to tell you that your manuscript has been accepted for publication in Journal of Cell Science, pending standard ethics checks.

© 2022. Published by The Company of Biologists under the terms of the Creative Commons Attribution License (https://creativecommons.org/licenses/by/4.0/).
